# Supplementary material for: Inflammatory factors and risk of lung adenocarcinoma: a Mendelian randomization study mediated by blood metabolites
Source: Front Endocrinol (Lausanne). 2024 Aug 27;15:1446863. doi: 10.3389/fendo.2024.1446863 (PMC11384989; doi:10.3389/fendo.2024.1446863)
Supplement: Supplementary file 1 [file DataSheet1.zip › supplementary files/S2/GCST90200304/forest.pdf]

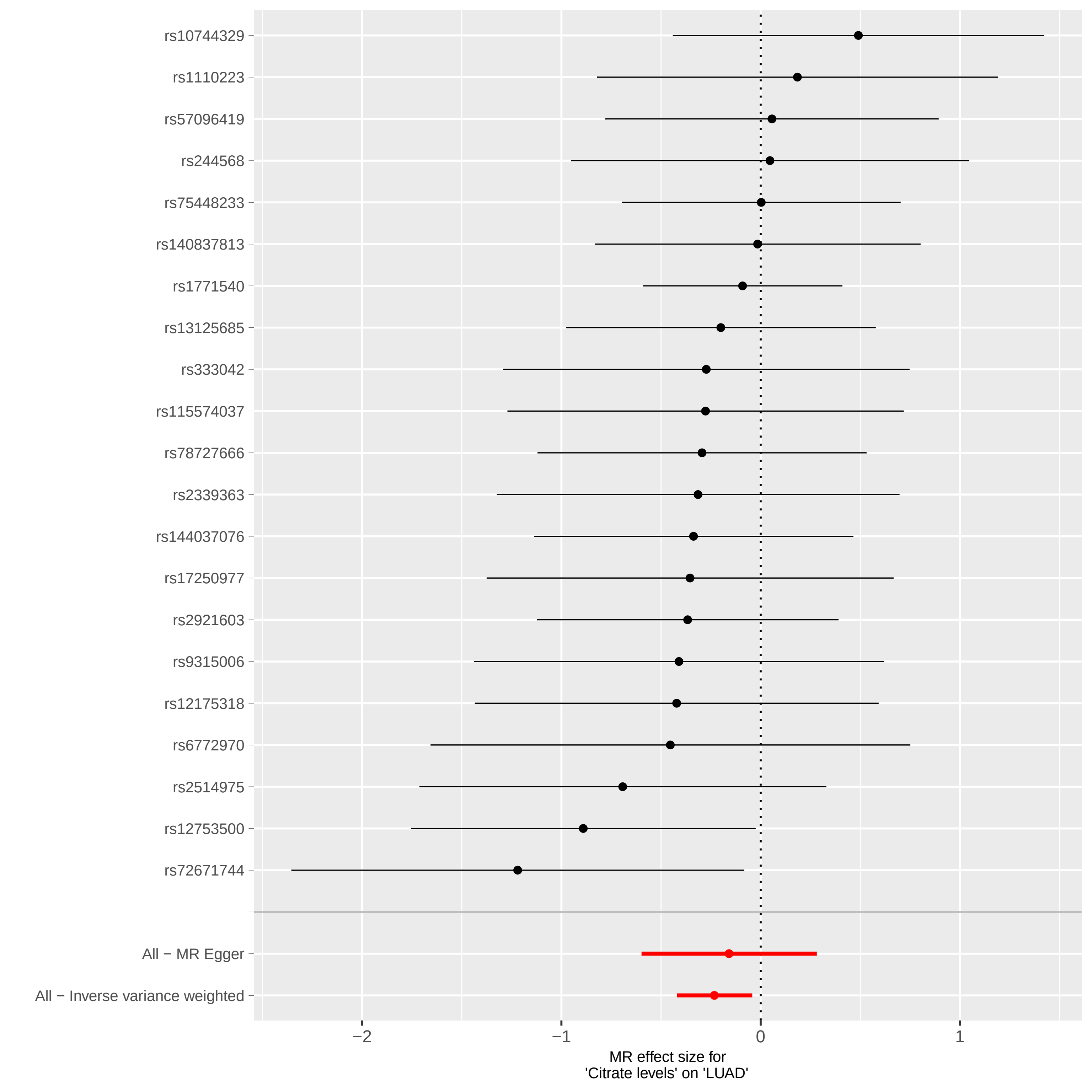
rs10744329
rs1110223
rs57096419
rs244568
rs75448233
rs140837813
rs1771540
rs13125685
rs333042
rs115574037
rs78727666
rs2339363
rs144037076
rs17250977
rs2921603
rs9315006
rs12175318
rs6772970
rs2514975
rs12753500
rs72671744
All − MR Egger
All − Inverse variance weighted
−2
−1
0
1
MR effect size for
'Citrate levels' on 'LUAD'
